# Method of Detecting Microorganisms on the Surface of Mandarin Fish Based on Hyperspectral and Information Fusion

**DOI:** 10.3390/foods14091468

**Published:** 2025-04-23

**Authors:** Tao Yuan, Yixiao Ma, Zuyu Guo, Yijian Wang, Liqin Kong, Yaoze Feng, Haopeng Liu, Liang Meng

**Affiliations:** 1 College of Engineering, Huazhong Agricultural University, Wuhan 430070, China; yuantao@mail.hzau.edu.cn (T.Y.); yijian.wang@webmail.hzau.edu.cn (Y.W.); kongliqin_hzau@163.com (L.K.); yaoze.feng@mail.hzau.edu.cn (Y.F.); liuhaopeng@mail.hzau.edu.cn (H.L.); 2 School of Electrical and Electronic Engineering, Wuhan Polytechnic University, Wuhan 430070, China; mayixiaomail@foxmail.com (Y.M.); 18755781031@163.com (Z.G.)

**Keywords:** hyperspectral technology, mandarin fish, microbes, non-destructive testing, freshness

## Abstract

Microorganisms play a key role in fish spoilage and quality deterioration, making the development of a rapid, accurate, and efficient technique for detecting surface microbes essential for enhancing freshness and ensuring the safety of mandarin fish consumption. This study focused on the total viable count (TVC) and *Escherichia coli* levels in the dorsal and ventral parts of fish, and we constructed a detection model using hyperspectral imaging and data fusion. The results showed that comprehensive and simplified models were successfully developed for quantitative detection across all wavelengths. The models performed best at predicting microbial growth on the dorsal side, with the RAW-CARS-PLSR model proving the most effective at predicting TVC and *E. coli* counts in that region. The RAW-PLSR model was identified as the optimal predictor of the *E. coli* concentration on the ventral side. A fusion model in the decision layer constructed using the Dempster–Shafer theory of evidence outperformed models relying solely on spectral or textural information, making it an optimal approach for detecting surface microbes in mandarin fish. The best prediction accuracy for dorsal TVC concentration achieved an Rp value of 0.9337, whereas that for ventral TVC concentration reached 0.8443. For the *E. coli* concentration, the optimal *R_p_* values were 0.8180 for the dorsal section and 0.8512 for separate analysis.

## 1. Introduction

Mandarin fish, classified within the order Perciformes, family Sinipercidae, subfamily Siniperchinae, and genus *Siniperca* (Basilewsky), is a freshwater species known for its delicate savory flesh. Characterized by relatively few bones and a broad, thick body, it is highly valued for its palatability and nutritional richness, containing over 20% protein per 100 g along with various trace elements, contributing to its significant economic value. The freshness of mandarin fish can directly affect their flavor, texture, and nutritional benefits, whereas deterioration can lead to toxicity and health risks primarily driven by microbial activity [1]. During the capture and postmortem phases, microbial interactions initiate biochemical processes that lead to rigor mortis, maturation, autolysis, and eventual decomposition in fish. Although the flesh remains fresh during rigor mortis and maturation, microbial infiltration accelerates the transition to autolysis and spoilage [2]. Thus, microbial activity is the primary determinant of decay, with bacterial counts and coliform levels serving as the key indicators of fish freshness.

Merchants often use freezing for storage, which can compromise the quality, making freshness assessment crucial. Current technologies for evaluating fish freshness include sensory evaluation, chemical indicator tests (e.g., pH levels, TVB-N content, K value, moisture, fat, and protein content), physical indicator tests (e.g., color, texture, and water-holding capacity), and biomimetic sensor technologies such as electronic noses and tongues [3]. However, traditional methods are inadequate for rapid, non-destructive evaluation and lack widespread application. Hyperspectral imaging (HSI) technology provides a noninvasive, rapid analytical approach that captures optical images that reflect both the internal chemical indicators and geometric features of fish samples. In a study by Zhou et al. [4], hyperspectral information was extracted using three different methods to simulate the growth of *Pseudomonas fluorescens*. The Baranyi model coupled with the Ratkowsky square root model (BRSR model) and the Huang model paired with the Ratkowsky square root model (HRSR model) were employed to directly construct growth simulations for *P*. *fluorescens* in pork using a one-step method [4]. The texture feature information in HSI as a key two-dimensional characteristic not only represents the grayscale spatial arrangement of samples but also provides insights into their quality. Gamilla et al. [5] converted color images obtained from machine vision into CIELAB values and used an analysis of variance to assess the freshness of different meat products. By integrating spectral and image texture data, this method simplifies complex information while simultaneously assessing both internal and external quality, enhancing the accuracy and stability of detection models, and significantly improving the non-destructive evaluation of fish meat [6].

The method proposed in this study integrates spectral analysis and information fusion to assess mandarin fish freshness, effectively addressing the limitations of traditional rapid non-destructive testing techniques. This approach provides an innovative and practical method to evaluate the freshness of mandarin fish in production settings. To verify the effectiveness of hyperspectral technology in the quantitative detection of surface microbial counts in mandarin fish, this study demonstrates that hyperspectral technology is effective, accurate, and highly adaptable for the quantitative detection of fish meat. These findings provide a strong theoretical foundation for the development of portable detection devices.

## 2. Materials and Methods

### 2.1. Acquisition of Mandarin Fish Samples

The experimental samples were obtained from the Yuehuo Li Life Plaza in Wuhan City, Hubei Province. The fish were culled by a blow to the head, and their viscera were removed. In a sterile environment, surgical tools were used to extract tissue samples from both the dorsal and ventral sides of the mandarin fish, carefully removing surface fat and connective tissue. The samples were precisely cut to 4.0 cm × 4.0 cm × 1.5 cm, sealed in sterile Petri dishes, and stored at a constant temperature of 4 °C in a biochemical incubator, with a total of 108 samples.

**Figure 1:**
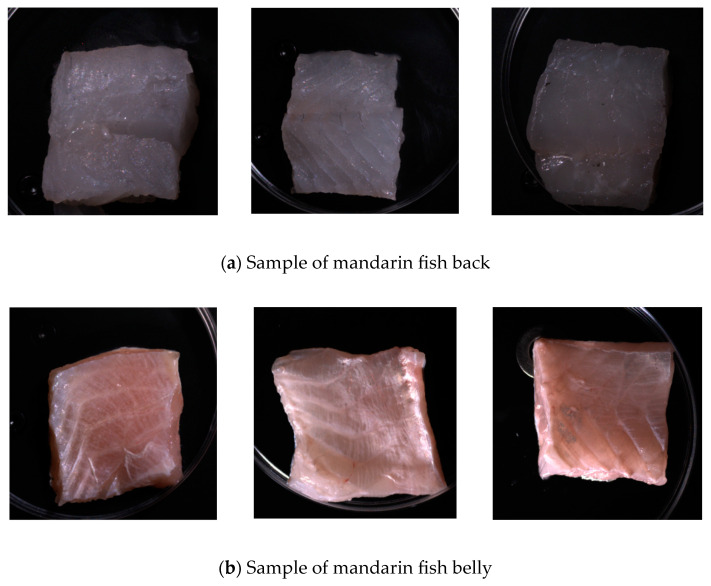
Back and abdominal samples of mandarin fish.

On days 0, 2, 3, 4, 5, and 6, six samples from both dorsal and ventral sections were collected for spectroscopic scanning. After scanning, the total microbial load and *Escherichia coli* count were measured according to national standards. The total microbial load was calculated using the GB4789.2-2022 standard [7], and the *E. coli* count was calculated using the GB 4789.3-2016 standard [8]. The experiment was conducted in three batches, each consisting of 36 dorsal and 36 ventral samples with 108 samples per batch. Figure 1 shows photographs of the dorsal and ventral samples, highlighting the differences in color, texture, and tissue structure. Therefore, this study focused on spectroscopic imaging and microbial counting tests for these distinct areas to determine the most effective sites for detecting surface microbial populations in mandarin fish [9].

### 2.2. Collection and Information Extraction of Hyperspectral Images of Mandarin Fish

**Figure 2:**
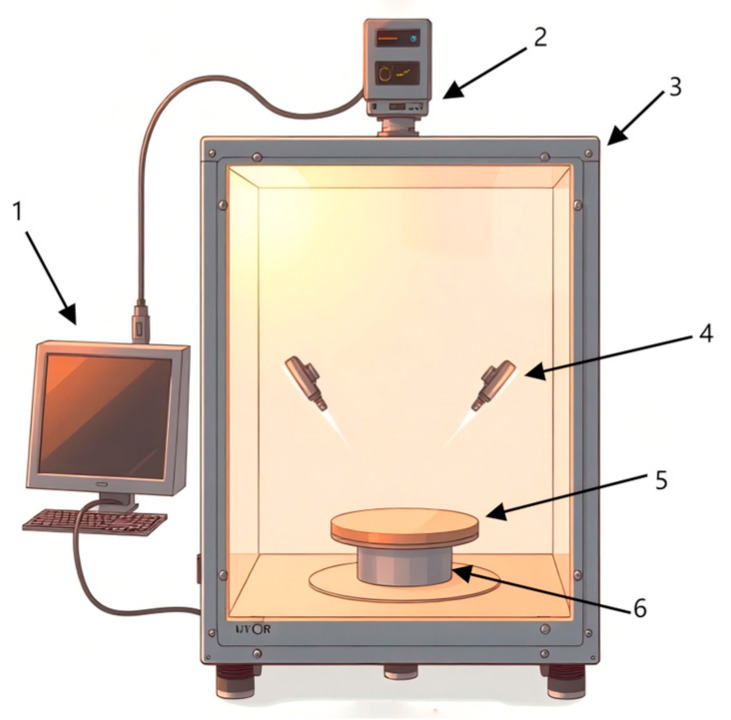
Hyperspectral image acquisition system. 1. Computer; 2. hyperspectral imager; 3. acquisition box; 4. halogen lighting source; 5. carrier stage; 6. high-precision electrically controlled movable elevating platform.

The experimental hyperspectral imaging system shown in Figure 2 consists of a hyperspectral imager (SPECIM, V10E, Helsiki, Finland), high-precision electronically controlled elevating platform (Beijing Zhuolihan Optical Instrument Co., Ltd., HSIA-TSA300-IMS, Beijing, China), collection box, cooling fans, halogen light sources, and a Lenovo ThinkPad computer (Beijing, China). The spectrometer captured wavelengths from 300 to 1100 nm with a spectral resolution of 2.8 nm.

**Figure 3:**
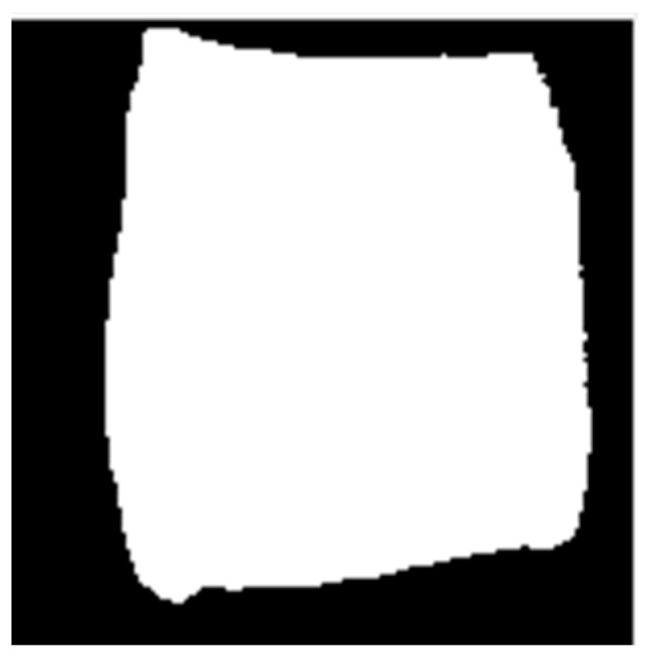
Mask image of the sample.

The hyperspectral imaging system captures images in a single acquisition, encompassing multiple sample details under varying adulteration ratios. Initially, the corrected images were segmented to extract individual spectral images of each sample. These segmented images then underwent region of interest (ROI) extraction to minimize background spectral interference. Following threshold segmentation, morphological operations such as dilation and erosion were applied to create a mask that accurately identified the ROI. Finally, the average spectrum of the sample was obtained from the masked localized area. Figure 3 illustrates the sample mask image, where the white area represents the spectral data extraction region, demonstrating the precise alignment of the mask with the sample.

### 2.3. Data Processing Methods

#### 2.3.1. Spectral Preprocessing Techniques

Owing to the complexities of spectral information and uncertainties arising from noise and errors during data collection, this study applied several spectral preprocessing techniques including smoothing [10], normalization [11], derivation [12], multiplicative scattering correction (MSC) [13], and standard normal variate transformation (SNV) [14].

#### 2.3.2. Extraction of Texture Feature Parameters

In this experiment, the gray level co-occurrence matrix (GLCM) was utilized to extract texture information from images [15], with the characteristic image data selected at 0°, 45°, 90°, and 135° in the hyperspectral images, encompassing six variables: mean, contrast, correlation, homogeneity, energy (also referred to as second moment), and entropy [16].

#### 2.3.3. Methodologies for Constructing Quantitative Models

Principal Component Regression (PCR) is a multivariate calibration method that combines Principal Component Analysis (PCA) with linear regression [17].

Partial Least Squares Regression (PLSR) utilizes a factor extraction approach that renders the new factorial variables mutually independent, adhering to the G-M conditions. The process begins with the establishment of a regression model between the dependent variables and the factors, which is then transformed into a regression model with independent variables [18].

The Backpropagation (BP) Neural Network is a multi-layer feedforward neural network that learns through an iterative error backpropagation process consisting of forward and backward propagation phases [19].

#### 2.3.4. Wavelength Selection Method

In this study, the Successive Projection Algorithm (SPA) [20], Competitive Adaptive Reweighting Algorithm (CARS) [21], and Genetic Algorithm (GA) [22] were employed to optimize predictive models for total viable count (TVC) and *E. coli* concentrations in the dorsal and ventral regions of mandarin fish.

#### 2.3.5. Information Fusion

Data layer fusion is the most basic level of integration and involves a direct combination of raw data. Although this approach preserves a large amount of information, it results in high temporal and spatial complexity, thereby reducing real-time capabilities. Moreover, the noise and interference inherent in raw data may negatively affect the results. This method is suitable for fusing information from homogeneous sensors but operates at low processing speeds [23].

Feature layer fusion represents an intermediate level of fusion in which information is condensed through preprocessing and feature extraction, thereby reducing noise and improving real-time processing capabilities. This approach preserves essential information and enhances noise resistance, making it suitable for the rapid computation and retention of critical feature information [24].

Decision-layer fusion represents an advanced level of fusion, in which data undergo preprocessing and modeling before integration. This approach optimizes the computational load and improves interference resistance, which may lead to information loss. In this study, the Dempster–Shafer theory was applied to further enhance the model performance [25].

## 3. Results and Discussion

### 3.1. Analysis of Microbial Spoilage in Mandarin Fish Samples

**Figure 4:**
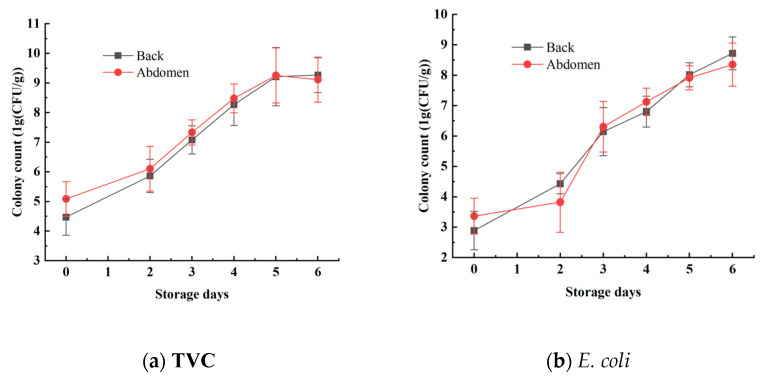
Changes in TVC and *E. coli* values on the surface of mandarin fish.

The TVC and *E. coli* concentrations in the mandarin fish samples were analyzed to effectively reflect the freshness of the samples. The statistical results for TVC and *E. coli* concentrations are shown in Figure 4 and Table 1. Using the plate counting method, the TVC concentration of the back ranged from 3.7475 log_10_ CFU/g to 7.4458 log_10_ CFU/g, whereas the TVC concentration of the abdomen ranged from 4.4024 log_10_ CFU/g to 10.6590 log_10_ CFU/g. The *E. coli* concentration of the back samples varied from 2.0000 log_10_ CFU/g to 9.4433 log_10_ CFU/g, and that of the abdominal samples ranged from 2.1761 log_10_ CFU/g to 9.4814 log_10_ CFU/g. These results show a reasonable range of variation, effectively reflecting the progression of mandarin fish samples from freshness to spoilage. These data are beneficial for establishing a stable and accurate predictive model [26].

During the period from days 0 to 2, the total bacterial count and *E. coli* numbers increased relatively slowly. However, from days 2 to 4, the growth rates of both total bacteria and *E. coli* increased significantly. Starting on day 5, as the storage time increased, the growth rate of the bacteria slowed again, which aligns with the typical patterns of bacterial growth curves. Additionally, the total bacterial count and *E. coli* numbers in the abdominal samples were generally higher than those in the back samples, indicating a higher susceptibility to spoilage in the abdominal samples. This difference is attributed to variations in tissue composition between the abdomen and the back [27].

Furthermore, national standards specify that aquatic products are considered to be spoiled when the total bacterial count exceeds 7 log_10_ CFU/g. This indicates that within the first 4 days (from day 0 to day 4), the mandarin fish samples remained in a usable state. However, after day 5, the freshness of the mandarin fish samples significantly declined, with microbial indicators beginning to exceed acceptable limits, rendering them inedible [28].

### 3.2. Sample Set Division

As shown in Table 2, 108 dorsal and 108 ventral samples of mandarin fish were subdivided at a ratio of 2:1, resulting in a training set and a prediction set. The dorsal and ventral samples included 72 and 36 specimens in the training and prediction sets, respectively. The SPXY algorithm was used to divide the sample set with the dorsal and ventral training sets employed for modeling, and the performance was subsequently assessed using the prediction set [29].

### 3.3. Detection of Microorganisms on the Surface of Mandarin Fish Based on Spectral Dimension Analysis

#### 3.3.1. Analysis of a Full-Wavelength Detection Model of Microorganisms on the Surface of Mandarin Fish

After preprocessing the spectral data of the dorsal region of the mandarin fish samples, full-wavelength regression prediction models for TVC and *E. coli* concentrations were established using Partial Least Squares Regression (PLSR), Principal Component Analysis (PCR), and Backpropagation (BP) Neural Networks. As shown in Table 3, both the TVC and *E. coli* concentration prediction models were based on RAW-PCR. For the TVC concentration, the training set yielded *R_c_* = 0.8642 and RMSEC = 0.8914, the cross-validation set yielded *R_cv_* = 0.6798 and RMSECV = 1.4540, and the prediction set yielded *R_p_* = 0.8402 and RMSEP = 1.0600. For the *E. coli* concentration, the training set achieved *R_c_* = 0.8871 and RMSEC = 1.0297, the cross-validation set showed *R_cv_* = 0.7788 and RMSECV = 1.4189, and the prediction set yielded *R_p_* = 0.8181 and RMSEP = 1.2500.

After preprocessing the spectral data of the abdominal region of the mandarin fish samples, full-wavelength regression prediction models for the TVC and *E. coli* concentrations were developed using PLSR, MLR, and BP Neural Networks. As shown in Table 4, the optimal prediction model for the TVC concentration in the abdominal region was the RAW-PLSR model, with the training set yielding *R_c_* = 0.9115 and RMSEC = 0.7259, the cross-validation set showing *R_cv_* = 0.7995 and RMSECV = 1.0751, and the prediction set resulting in *R_p_* = 0.7706 and RMSEP = 1.0797. Similarly, the best prediction model for the *E. coli* concentration was the RAW-PLSR model, with the training set achieving *R_c_* = 0.9486 and RMSEC = 0.6724, cross-validation set showing *R_cv_* = 0.7746 and RMSECV = 1.3626, and prediction set yielding *R_p_* = 0.7802 and RMSEP = 1.2725.

#### 3.3.2. Simplified Model Analysis of Surface Microorganism Detection of Mandarin Fish

As shown in Table 4, both the dorsal and ventral sample groups demonstrated satisfactory modeling performance when raw data were used to construct predictive models for the TVC and *E. coli* concentrations. Consequently, this study aimed to streamline the model construction process by selecting characteristic wavelengths from the original data using techniques such as CARS, GA, and SPA for model simplification.

**Figure 5:**
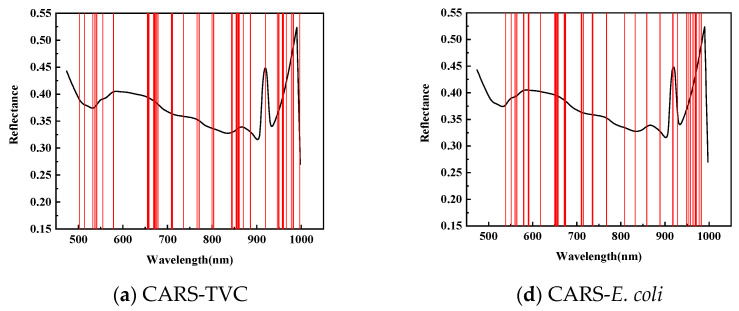

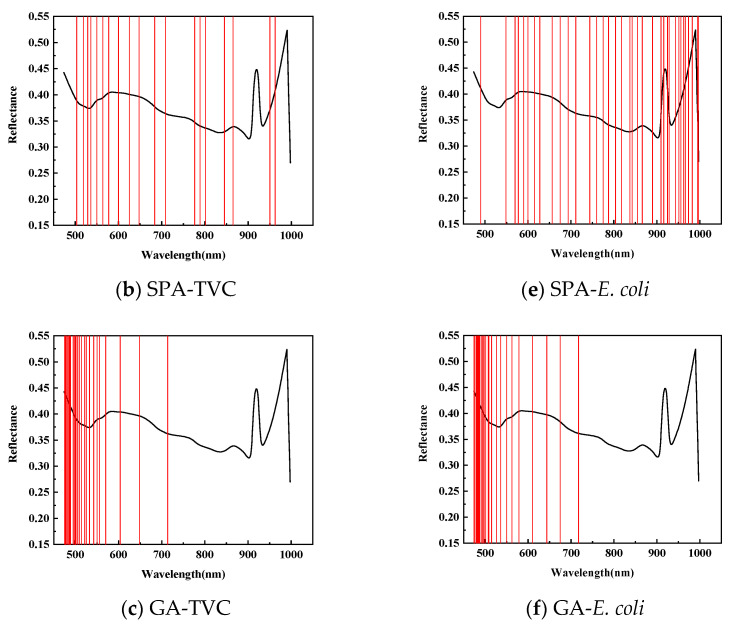
Distribution of characteristic wavelengths predicted for TVC and *E. coli* values in SPA-screened back samples.

Figure 5 illustrates the application of Competitive Adaptive Reweighted Sampling (CARS), the Successive Projection Algorithm (SPA), and the Genetic Algorithm (GA) to select the feature wavelengths from dorsal samples of mandarin fish to evaluate the total viable count (TVC) concentration. CARS identified 47 characteristic wavelengths, primarily within the 840–860 and 950–1000 nm ranges, with fewer in the 550–650 nm spectrum during 50 Monte Carlo samplings. The SPA selected 19 characteristic wavelengths, mainly distributed within the 710–780 and 860–945 nm ranges, with fewer in the 950–1000 nm range, and a more uniform presence across the 500–710 nm spectrum. After 20 evaluations, the GA identified 26 characteristic wavelengths, predominantly in the 475–550 nm range, with minimal presence across the 600–1000 nm spectrum.

To predict the *E. coli* concentration in the dorsal samples of mandarin fish, CARS identified 39 characteristic wavelengths, primarily within the 650–710 and 920–970 nm spectra, with fewer distributed between 470 and 550 nm during 50 Monte Carlo samplings. The SPA selected 36 characteristic wavelengths with a relatively even distribution while being similarly sparse in the 470–550 nm range. After 20 evaluations, the GA identified 26 characteristic wavelengths concentrated in the 470–710 nm range, particularly within the 470–550 nm range, with almost no detection in the 720–1000 nm spectrum, exhibiting distinct selection patterns compared with CARS and the SPA.

As shown in Table 5, an optimized model for correlating TVC and *E. coli* concentrations in the dorsal muscles of mandarin fish was developed using the feature wavelengths selected by the CARS method. This model was termed RAW-CARS-PLSR and employed PLSR, thereby demonstrating its superior accuracy and predictive performance. For the TVC concentration, the training set achieved a correlation coefficient (*R_c_*) of 0.9226 with a standard error of calibration (RMSEC) of 0.7541, the cross-validation set had an *R_cv_* of 0.8299 with a standard error of cross-validation (RMSECV) of 1.1058, and the prediction set yielded an *R_p_* of 0.8437 with a standard error of prediction (RMSEP) of 0.9512. For the *E. coli* concentration, the cross-validation set achieved an *R_cv_* of 0.8576 with an RMSECV of 1.1635, whereas the prediction set recorded an *R_p_* of 0.7632 with an RMSEP of 1.4046. Compared with other wavelength selection methods, the CARS-based model demonstrated higher correlation coefficients and lower errors in both the training and prediction sets. This is consistent with the findings of Baek et al. (2020), who demonstrated that the modeling performance of the feature wavelength selection method was overall superior [30].

**Figure 6:**
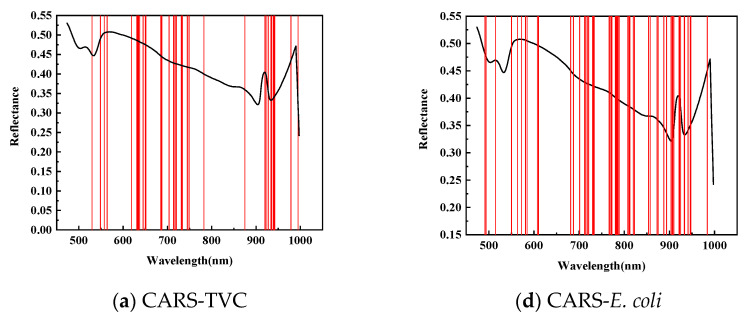

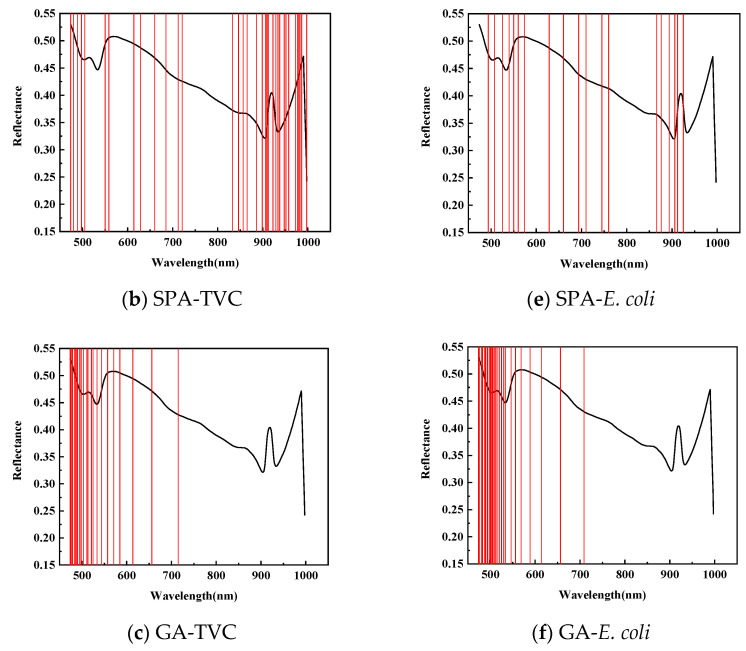
Prediction of characteristic wavelength distribution using TVC and *E. coli* values of abdominal samples screened by CARs.

As shown in Figure 6, for feature wavelength selection in mandarin fish abdominal samples to predict TVC concentration, CARS identified 38 characteristic wavelengths during 50 Monte Carlo samplings, primarily between 620–750 and 920–950 nm, with fewer wavelengths in the 420–550 and 780–875 nm ranges. The SPA selected 36 characteristic wavelengths, mainly concentrated between 820–1000 and 470–710 nm, with almost no distribution between 720 and 830 nm. After 20 evaluations, the GA identified 24 characteristic wavelengths predominantly within the 470–550 nm range, with minimal presence between 710 and 1000 nm. This demonstrates a divergence from the CARS and SPA results and indicates insufficient modeling performance.

As shown in Table 6, to predict the *E. coli* concentration in mandarin fish abdominal samples, CARS identified 58 characteristic wavelengths during 50 Monte Carlo samplings, primarily distributed between 700 and 950 nm, with fewer in the 420–550 nm and 610–700 nm ranges. The SPA selected 19 characteristic wavelengths, mainly between 490–570 and 860–930 nm, with a sparse distribution between 760 and 860 nm. The GA applied to dorsal samples for *E. coli* concentration prediction identified 28 characteristic wavelengths primarily concentrated in the 470–550 nm range, with almost no wavelengths detected between 710 and 1000 nm.

When different wavelength selection methods were adopted to develop simplified models for predicting TVC and *E. coli* concentrations in the abdomen of mandarin fish, the PLSR model based on Competitive Adaptive Reweighted Sampling (RAW-CARS-PLSR) demonstrated the best performance. For the TVC concentration, the training set achieved *R_c_* = 0.9437 and RMSEC = 0.5836. The cross-validation set showed *R_cv_* = 0.8935 and RMSECV = 0.8037, and the prediction set yielded *R_p_* = 0.8464 and RMSEP = 0.9474. For *E. coli* concentration, the training set recorded *R_c_* = 0.9516 and RMSEC = 0.6528, the cross-validation set achieved *R_cv_* = 0.8868 and RMSECV = 0.9956, and the prediction set resulted in *R_p_* = 0.7239 and RMSEP = 1.4035.

#### 3.3.3. Comparison and Analysis of Full Wavelength Spectral Data of Different Detection Sites and Simplified Models

As shown in Table 7, a comparison of predictive models for TVC on the dorsal and ventral surfaces of mandarin fish revealed that the model for the ventral section outperformed that for the dorsal area, indicating superior accuracy in forecasting the total microbial count on the fish surface. The utilization of untreated raw data produced the most effective model performance, with the Polymerase Chain Reaction (PCR) model demonstrating notable effectiveness in quantitative predictions. Additionally, the application of CARS to select characteristic wavelengths further enhances its efficacy.

For the models predicting of *E. coli* concentration, the ventral section demonstrated superior modeling performance compared with the dorsal section, whereas the dorsal models exhibited a smaller margin of error in predictions. The optimal preprocessing method, involving the use of raw data with the PCR model and CARS, proved to be the most effective for constructing predictive models. This is consistent with the findings of Ma et al. [31], who indicated that spectral information varied across different parts of fish fillets.

### 3.4. Detection of Surface Microorganisms of Mandarin Fish Based on Texture Information

#### 3.4.1. Mandarin Fish Surface Microbial Detection Model Based on Feature Band Texture Analysis

For texture feature extraction from hyperspectral images of mandarin fish, the gray level co-occurrence matrix (GLCM) method was used to derive six texture parameters, including the mean, contrast, correlation, homogeneity, energy, and entropy, from the samples of the main body and belly regions. These parameters served as variables for constructing a microbial detection model based on surface texture information. During feature extraction, gray-level quantization was set to 16 levels, with a distance (d) of 1. GLCMs were computed in four orientations, that is, 0°, 45°, 90°, and 135°, and the averages across these directions were used as textural feature parameters. To normalize the data, the sum of all parameters was calculated, and each parameter was divided by this sum, scaling the values within the range [0, 1].

As demonstrated in Section 3.4, the CARS method significantly improved the accuracy of model predictions, making it the most effective among the three feature wavelength selection methods. This section describes the application of the CARS method. The spectral data from the 472.5 to 997.5 nm bands were initially divided into 20 equal subsets, followed by the development of 21 subsets and a full-spectrum microbial PLSR detection model. The RMSEs of the subset-based PLSR models were compared with those of the full-spectrum model, and the four subsets with the lowest RMSE, each less than that of the full-spectrum model, were selected. CARS was then applied to these four subsets to further refine the bands, reducing the variable numbers, eliminating the redundant information, and extracting the optimal texture features for modeling.

Using this methodology, the feature wavelengths identified for the dorsal TVC prediction were 656.25, 658.75, 670, 673.75, 675, 858.75, and 861.25 nm, with the corresponding texture features producing 48 texture variables per sample. For the ventral TVC prediction, the feature wavelengths were 631.25, 633.75, 637.5, 715, 718.75, 733.75, 935, 941.25, and 942.5 nm, resulting in 54 texture variables per sample.

For the dorsal *E. coli* concentration prediction, the extracted feature wavelengths were 653.75, 656.25, 673.75, 948.75, 952.5, 966.25, and 977.5 nm, yielding 42 texture variables per sample. For the ventral *E. coli* prediction, the feature wavelengths were 712.5, 718.75, 721.25, 770, 771.25, 780, 781.25, 872.5, 875, 925, and 932.5 nm, resulting in 66 texture variables per sample. The modeling outcomes for microbial concentration predictions based on texture features from both anatomical regions are presented in Table 8.

As shown in Table 8, when using the characteristic band texture analysis method to build the TVC concentration prediction model for the surface of mandarin fish, the PCR model demonstrated the best performance. For the dorsal prediction model, the training set achieved an *R_c_* of 0.7324 with an RMSEC of 1.1411, the cross-validation set showed *R_cv_* = 0.6471 and RMSECV = 0.5138, and the prediction set yielded *R_p_* = 0.7168 and RMSEP = 1.3571. For the abdominal prediction model, the training set recorded *R_c_* = 0.7535 and RMSEC = 1.4471, the cross-validation set achieved *R_cv_* = 0.6728 and RMSECV = 0.5047, and the prediction set resulted in *R_p_* = 0.7104 and RMSEP = 1.2748. A comparison of the two anatomical regions indicated that the abdominal model outperformed the dorsal model, which is consistent with the results of the previous model based on spectral dimensions.

When constructing an *E. coli* concentration prediction model on the surface of mandarin fish using texture analysis of feature bands, the PCR model demonstrated the best performance. For the dorsal model, the training set achieved an *R_c_* of 0.7221 with RMSEC = 1.4241, the cross-validation set showed *R_cv_* = 0.6418 and RMSECV = 1.3987, and the prediction set yielded *R_p_* = 0.7097 and RMSEP = 1.4635. For the abdominal model, the training set recorded *R_c_* = 0.7549 and RMSEC = 1.3341, the cross-validation set achieved *R_cv_* = 0.6646 and RMSECV = 1.3352, and the prediction set resulted in *R_p_* = 0.7401 and RMSEP = 1.3789.

However, the prediction model based on feature band texture analysis has certain limitations compared with the spectral-dimension-based model, resulting in a slightly lower overall modeling performance.

#### 3.4.2. Regression Model Based on Principal Component Image Texture Analysis

This study applied a Principal Component Analysis (PCA) to reduce the dimensionality of hyperspectral images from the dorsal and ventral regions of mandarin fish, selecting the first three principal component images (PC1, PC2, and PC3), which accounted for 95.2%, 2.9%, and 0.9% of the cumulative spectral variance, respectively, totaling over 98%. The texture features of these three principal components were then extracted using the gray level co-occurrence matrix (GLCM), resulting in 18 texture feature parameters. The training and testing sets were structured into matrices of 18 (feature count) × 72 (sample count) and 18 (feature count) × 36 (sample count), respectively. The results are presented in Table 9.

As shown in Table 9, when predicting TVC concentration on the surface of mandarin fish using the texture analysis method of principal component images, the PCR model demonstrated the best performance among the models for both the dorsal and abdominal regions. For the dorsal model, the training set achieved a correlation coefficient *R_c_* of 0.7318 with RMSEC = 1.3998, the cross-validation set showed *R_cv_* = 0.6654 and RMSECV = 1.517, and the prediction set yielded *R_p_* = 0.7074 and RMSEP = 1.4122. For the abdominal model, the training set recorded an *R_c_* of 0.7633 with RMSEC = 1.3323, the cross-validation set achieved *R_cv_* = 0.6943 and RMSECV = 1.4378, and the prediction set resulted in *R_p_* = 0.7394 and RMSEP = 1.3875.

When predicting *E. coli* concentrations in the dorsal and abdominal regions of mandarin fish using principal component image texture analysis, the PCR model outperformed the other models. For the dorsal region, the training set achieved *R_c_* = 0.7079 and RMSEC = 1.2064, the cross-validation set showed *R_cv_* = 0.6383 and RMSECV = 1.4211, and the prediction set yielded *R_p_* = 0.6831 and RMSEP = 1.3273. The PCR model performed slightly better, with the training set recording *R_c_* = 0.7635 and RMSEC = 1.1138, the cross-validation set achieving *R_cv_* = 0.6896 and RMSECV = 1.4699, and the prediction set resulting in *R_p_* = 0.7459 and RMSEP = 1.2511.

Overall, the prediction model based on the PCA of image textures outperformed the model using feature band texture analysis but remained inferior to the spectral dimension model. Compared with the optimal models presented in Section 3.4 and Section 3.5, the correlation coefficients *R_c_*, *R_cv_*, and *R_p_* were significantly lower, indicating that the texture information was less effective at predicting the microbial counts on the surface of mandarin fish than the spectral dimension data. The research by Liu et al. [32] also supports this viewpoint, demonstrating that when studying salted meat, there is a significant difference in the accuracy of models established using the two types of information, with spectral information performing notably better. Although the texture information primarily reflected the external quality of the samples, the spectral data provided a more comprehensive representation of internal quality. Because the decay process progressed from the outside inward, the spectral data offered a distinct advantage for predicting microbial counts on the fish surface.

### 3.5. Surface Microbial Detection of Mandarin Fish Based on Information Fusion

#### 3.5.1. Surface Microbial Detection Model of Mandarin Fish Based on Feature Layer Fusion

Before developing a predictive model for TVC concentration on the surface of mandarin fish using feature layer fusion, differences in the physical significance and numerical range of spectral and textural data were considered. To improve the accuracy and effectiveness of the model, data normalization was applied by adjusting both the spectral and textural features to a uniform range of [0, 1]. After normalization, feature fusion was performed to ensure data comparability, thereby facilitating the construction of a more stable model. The results of the microbial detection model based on feature layer fusion are presented in Table 10.

As shown in Table 10, the TVC concentration PCR model developed using feature-layer fusion demonstrated advantages over models based only on textural information, as reflected by the increased *R_c_*, *R_cv_*, and *R_p_* values. However, it remained slightly inferior to the models that used only spectral data, primarily owing to the suboptimal performance of texture information, indicating a limitation in the representativeness of texture feature extraction. In terms of stability, the small discrepancy between RMSEC, RMSECV, and RMSEP suggested that while the accuracy decreased, the stability improved.

When constructing the *E. coli* concentration prediction model, a trend similar to that of the TVC concentration prediction model was observed. Compared to the models based on spectral dimensions, the coefficients of determination (*R_c_*, *R_cv_*, and *R_p_*) decreased, whereas the root mean square errors (RMSEC, RMSECV, and RMSEP) increased, indicating that feature-layer fusion did not improve the modeling effectiveness compared to using a single information source.

#### 3.5.2. Surface Microbial Detection Model of Mandarin Fish Based on Decision-Level Fusion

Using the optimal predictive model results from Section 2.2 for the TVC concentrations and *E. coli* values in the dorsal and ventral samples of mandarin fish, combined with the superior texture-based predictive results from Section 2.3, these inputs were integrated into a detection model based on decision-level fusion, resulting in the development of a microbial detection model for the surface of mandarin fish.

In the decision-level fusion process of spectral and texture information from the dorsal and ventral samples of mandarin fish, the first step involved combining the optimal predictive outcomes from both channels into a two-dimensional decision vector, which was used as the input for modeling using the PCR method. Next, a second decision-level fusion approach was applied using the Dempster–Shafer evidence theory algorithm to convert the two-dimensional decision vector into a one-dimensional decision vector, which was again used as the input for PCR modeling. Finally, a comparative analysis was conducted to evaluate the modeling performance of these two decision-level fusion techniques.

As shown in Table 11, the TVC concentration prediction model developed through decision-level fusion exhibited strong overall stability, with the decision coefficients (*R_c_*, *R_cv_*, and *R_p_*) closely aligned and predominantly higher, indicating consistent performance across datasets. Additionally, the root mean square errors (RMSEC, RMSECV, and RMSEP) demonstrated minimal differences and were notably lower than those of the models using only spectral or textural information. The decision-level fusion model for predicting *E. coli* concentrations in mandarin fish also demonstrated an overall favorable modeling outcome. Although the model constructed through direct merging did not exhibit a clear advantage over the spectral-dimension model in terms of the decision coefficients (*R_c_* and *R_p_*), the RMSEC, RMSECV, and RMSEP values were comparable, reflecting stable and reliable performance. Ahmed et al. [33] suggest that information fusion technology involves associating or integrating data and information from single or multiple channels in various ways, enabling more accurate acquisition of target information. It can also compensate for the limitations of the data that a single channel cannot obtain. Although this increases system complexity, it often leads to significant performance improvements.

The performance of the prediction model was further enhanced by applying the D-S evidence theory algorithm, which improved the stability and accuracy of the TVC concentration model based on decision-level fusion. This demonstrates that combining information from different sources can effectively enhance predictive capabilities. The optimal TVC concentration prediction model for the dorsal region of mandarin fish achieved *R_c_* = 0.9516 and RMSEC = 0.9315, a cross-validation set *R_cv_* = 0.7469 and RMSECV = 0.8822, and a prediction set *R_p_*= 0.9337 and RMSEP = 0.8389. For the abdominal TVC concentration model, the results were *R_c_* = 0.9401 and RMSEC = 0.7387. The cross-validation set achieved *R_cv_* = 0.8903 and RMSECV = 0.9026, and the prediction set obtained Rp = 0.8443 and RMSEP = 1.1464. The optimal *E. coli* concentration prediction model for the dorsal region achieved *R_c_* = 0.8897 and RMSEC = 0.9699, the cross-validation set achieved *R_cv_* = 0.8786 and RMSECV = 1.0146, and the prediction set obtained *R_p_*= 0.8180 and RMSEP=1.2501. For the abdominal region, the *E. coli* model results were *R_c_* = 0.9469 and RMSEC = 0.7443. The cross-validation set achieved *R_cv_* = 0.8969 and RMSECV = 0.8995, and the prediction set obtained *R_p_*= 0.8512 and RMSEP = 1.1224. These results indicate that the decision-level fusion model is the optimal approach for predicting microbial concentrations in mandarin fish.

## 4. Conclusions

A novel model utilizing hyperspectral imaging technology was proposed to detect the concentrations of TVC and *E. coli* in mandarin fish, predicting the concentrations on both the dorsal and ventral sides during storage from 0 to 6 days. The analytical results indicated that the predictive model constructed using the D-S evidence theory for decision-level fusion was significantly more effective than models based only on spectral or texture information. Decision-level fusion proved to be the optimal method for predicting microbial counts on fish surfaces. In conclusion, the application of hyperspectral imaging technology for assessing the freshness of mandarin fish is both feasible and reliable. However, further exploration and research are still necessary, particularly for the detection of surface microorganisms based on texture information, as the predictive performance of the established models remains relatively weak and requires algorithmic enhancement to improve accuracy. Additionally, this study focused solely on developing a hyperspectral-based model for predicting microbial quantities; however, the underlying detection mechanisms remain unclear and warrant further investigation. In the future, it may also be possible to develop a non-destructive detection device based on hyperspectral technology for microbial quantification, making it more suitable for practical industrial applications.

## Figures and Tables

**Table 1:** Basic statistical results of TVC and *E. coli* concentrations in mandarin fish.

Location	Microbial Indicator	Minimum Value	The Maximum Value	Average Value	Standard Deviation
Back	TVC	3.7475	10.6551	7.4458	1.8965
	*E. coli*	4.4024	10.6590	7.6892	1.7456
Abdomen	TVC	2.0000	9.4433	6.1308	2.2150
	*E. coli*	2.1761	9.4814	6.2449	2.1027

**Table 2:** Statistical results of microorganism quantity on the surface of mandarin fish after sample set division.

Location	Microbial Indicator	Training Set			Test Set		
		Variation Range	Mean	Standard Deviation	Variation Range	Mean	Standard Deviation
Back	TVC	3.74~10.65	7.47	1.96	4.05~9.98	7.38	1.77
	*E. coli*	2.00~9.44	6.05	2.31	2.39~9.17	6.29	2.17
Abdomen	TVC	4.40~10.65	7.61	1.76	4.46~10.49	7.85	1.69
	*E. coli*	2.17~9.48	6.12	2.12	2.65~9.00	6.50	2.03

**Table 3:** Results of prediction models for TVC and *E. coli* values in mandarin fish backs using different pretreatment methods.

Microbial Indicator	Model	Pretreatment Method	*R_c_*	RMSEC	*R_cv_*	RMSECV	*R_p_*	RMSEP	ΔE
**TVC**	PLSR	RAW	0.8403	0.9605	0.6515	1.5041	0.8358	1.0734	0.1129
		SG	0.8398	0.9618	0.6517	1.5037	0.8350	1.0757	0.1139
		VN	0.8230	1.0060	0.6140	1.5648	0.8067	1.1552	0.1492
		1stDe	0.8830	0.9174	0.5750	1.6220	0.7593	1.1530	0.2356
		MSC	0.8335	1.0799	0.6549	1.4981	0.7740	1.1219	0.0420
		SNV	0.8554	0.9177	0.6124	1.5672	0.8437	1.0492	0.1315
	PCR	RAW	0.8642	0.8914	0.6798	1.4540	0.8402	1.0600	0.1686
		SG	0.8643	0.8910	0.6787	1.4558	0.8407	1.0587	0.1677
		VN	0.7942	1.0767	0.6164	1.5611	0.7675	1.2531	0.1764
		1stDe	0.7650	1.2590	0.5156	1.6987	0.6294	1.3769	0.1179
		MSC	0.8394	1.0624	0.6867	1.4412	0.6756	1.3062	0.2438
		SNV	0.8499	0.9335	0.6515	1.5435	0.8299	1.0900	0.1565
	BP	RAW	0.7794	0.8166	-	-	0.5667	1.1920	0.3754
		SG	0.7219	0.8143	-	-	0.5570	1.1489	0.3346
		VN	0.6751	0.9616	-	-	0.6014	1.1559	0.1943
		1stDe	0.7784	0.7387	-	-	0.6762	1.0191	0.2804
		MSC	0.7529	0.8578	-	-	0.4339	1.3035	0.4457
		SNV	0.6848	0.8576	-	-	0.8358	1.2131	0.3555
***E. coli***	PLSR	RAW	0.8721	1.0914	0.7455	1.5078	0.8054	1.2882	0.1968
		SG	0.8936	1.0012	0.7648	1.4575	0.8180	1.2501	0.2489
		VN	0.8716	1.0936	0.7464	1.5057	0.7884	1.3371	0.2435
		1stDe	0.9155	0.8972	0.6580	1.7036	0.7465	1.4463	0.5491
		MSC	0.8710	1.0960	0.7329	1.5391	0.6218	1.7024	0.6064
		SNV	0.8703	1.0986	0.6993	1.6173	0.7756	1.3719	0.2733
	PCR	RAW	0.8871	1.0297	0.7788	1.4189	0.8181	1.2500	0.2203
		SG	0.8867	1.0313	0.7775	1.4228	0.8205	1.2423	0.2110
		VN	0.8720	1.0919	0.7589	1.4733	0.7838	1.3499	0.2580
		1stDe	0.7844	1.3836	0.5208	1.9315	0.6472	1.6569	0.2733
		MSC	0.8811	1.0549	0.7636	1.4608	0.6715	1.6107	0.5558
		SNV	0.8746	1.0814	0.7331	1.5387	0.7824	1.3536	0.2722
	BP	RAW	0.9027	0.6400	-	-	0.7407	1.1924	0.5524
		SG	0.8523	0.778	-	-	0.8091	1.0432	0.2652
		VN	0.5914	1.1994	-	-	0.4948	1.5425	0.3431
		1stDe	0.8452	0.7949	-	-	0.5394	1.4945	0.6996
		MSC	0.8795	0.7077	-	-	0.5176	1.5187	0.8110
		SNV	0.7806	0.9298	-	-	0.8054	1.5098	0.5800

**Table 4:** Results of prediction models for abdominal TVC and *E. coli* values of mandarin fish established using different pretreatment methods.

Microbial Indicator	Model	Pretreatment Method	$R_{c}$	RMSEC	$R_{cv}$	RMSECV	*R_p_*	RMSEP	ΔE
TVC	PLSR	RAW	0.9115	0.7259	0.7995	1.0751	0.7706	1.0797	0.3538
		SG	0.9169	0.7043	0.8043	1.0635	0.7422	1.1352	0.4309
		VN	0.9008	0.7665	0.7675	1.1474	0.7159	1.1828	0.4163
		1stDe	0.8928	0.7949	0.7720	1.1375	0.7950	1.0274	0.2325
		MSC	0.8900	0.8047	0.6183	1.4067	0.6938	1.2200	0.4153
		SNV	0.8994	0.7705	0.7410	1.2018	0.7420	1.1356	0.3651
	PCR	RAW	0.8996	0.7707	0.7992	1.0756	0.7987	1.0194	0.2487
		SG	0.9000	0.7693	0.7994	1.0743	0.7836	1.0523	0.2830
		VN	0.8841	0.8248	0.7563	1.1709	0.7312	1.1556	0.3308
		1stDe	0.8730	0.8606	0.7858	1.1069	0.7974	1.0222	0.1616
		MSC	0.6888	1.2795	0.5275	1.5205	0.5818	1.3778	0.0983
		SNV	0.8843	0.8239	0.7549	1.1739	0.7406	1.1383	0.3144
	BP	RAW	0.8350	0.6474	-	-	0.7662	0.8889	0.2415
		SG	0.9180	0.4666	-	-	0.6826	1.0109	0.5443
		VN	0.7625	0.7614	-	-	0.7243	0.9537	0.1923
		1stDe	0.9066	0.4965	-	-	0.6113	1.0947	0.5982
		MSC	0.7842	0.7301	-	-	0.7706	0.9038	0.1737
		SNV	0.9115	0.8868	-	-	0.7422	1.0955	0.2087
*E. coli*	PLSR	RAW	0.9486	0.6724	0.7746	1.3626	0.7802	1.2725	0.9130
		SG	0.8930	0.9559	0.7355	1.4597	0.6266	1.5854	0.3166
		VN	0.8514	1.1143	0.6947	1.5498	0.6430	1.5581	0.4438
		1stDe	0.8927	0.9573	0.7610	1.3978	0.7276	1.3954	0.4381
		MSC	0.6640	1.5887	0.5154	1.8466	0.5948	1.7010	0.1123
		SNV	0.8012	1.2712	0.4941	1.8733	0.4604	1.8059	0.5347
	PCR	RAW	0.8885	0.9746	0.7560	1.4104	0.7633	1.3323	0.3577
		SG	0.8897	0.9699	0.7620	1.3954	0.7549	1.3341	0.3642
		VN	0.8576	1.0926	0.7075	1.5227	0.7233	1.4048	0.3122
		1stDe	0.8786	1.0146	0.7648	1.3882	0.6972	1.4582	0.4436
		MSC	0.6037	1.6940	0.4994	1.8668	0.5856	1.7100	0.0160
		SNV	0.8500	1.1193	0.5873	1.7440	0.6168	1.6011	0.4818
	BP	RAW	0.8216	0.8075	-	-	0.5780	1.3555	0.5480
		SG	0.8281	0.7942	-	-	0.6767	1.2230	0.4288
		VN	0.7986	0.8527	-	-	0.6538	1.2568	0.4041
		1stDe	0.9209	0.5522	-	-	0.5718	1.3628	0.8106
		MSC	0.7699	0.9041	-	-	0.7019	1.1832	0.2791
		SNV	0.8101	0.8305	-	-	0.6003	1.3285	0.4980

**Table 5:** Simplified models of TVC and *E. coli* values in back samples using different wavelength selection methods for RAW pretreatment.

Index	Wavelength Selection Method	Characteristic Wavelength (nm)	Model	$R_{c}$	RMSEC	*R_cv_*	RMSECV	*R_p_*	RMSEP
TVC	CARS	47	PLSR	0.9226	0.7541	0.8299	1.1058	0.8437	0.9512
			PCR	0.9186	0.7723	0.8398	1.0761	0.8344	0.9765
			BP	0.8688	0.6453	-	-	0.7476	0.9609
	SPA	19	PLSR	0.8871	0.9024	0.7758	1.2507	0.8737	0.7721
			PCR	0.8332	1.0809	0.6393	1.5244	0.8737	0.7721
			BP	0.8570	0.6717	-	-	0.6567	1.0911
	GA	26	PLSR	0.7981	1.1779	0.5854	1.6072	0.6639	1.3250
			PCR	0.8033	0.3860	1.2464	0.3860	0.6884	1.2851
			BP	0.6624	0.9764	-	-	0.6525	1.0963
***E. coli***	CARS	39	PLSR	0.9309	0.8147	0.8576	1.1635	0.7632	1.4046
			PCR	0.9233	0.8569	0.8528	1.1816	0.7663	1.3966
			BP	0.8213	0.8485	-	-	0.6129	1.4049
	SPA	36	PLSR	0.8872	1.0293	0.7617	1.4658	0.7421	1.1355
			PCR	0.8759	1.0764	0.7677	1.4498	0.7421	1.1355
			BP	0.8608	0.741	-	-	0.6765	1.3072
	GA	26	PLSR	0.7592	1.4520	0.6130	1.7874	0.7324	1.4882
			PCR	0.7929	1.3595	0.6284	1.7598	0.7982	1.3094
			BP	0.7922	0.9077	-	-	0.7318	1.2097

**Table 6:** Simplified models of abdominal TVC and *E. coli* values established using different wavelength selection methods under RAW pretreatment.

Index	Wavelength Selection Method	Characteristic Wavelength (nm)	Model	$R_{c}$	RMSEC	*R_cv_*	RMSECV	$R_{p}$	RMSEP
TVC	CARS	38	PLSR	0.9437	0.5836	0.8935	0.8037	0.8464	0.9474
			PCR	0.9376	0.6136	0.8882	0.8222	0.7517	1.1172
			BP	0.8744	0.5710	-	-	0.7015	0.9818
	SPA	36	PLSR	0.8571	0.9090	0.7367	1.2102	0.7635	1.4038
			PCR	0.8642	0.8880	0.7477	1.1885	0.7635	1.4038
			BP	0.8700	0.5802	-	-	0.6829	1.0106
	GA	24	PLSR	0.8218	1.005	0.7199	1.2423	0.8168	0.9774
			PCR	0.8162	1.0197	0.7206	1.2410	0.8052	1.0046
			BP	0.8398	0.6388	0.8935	-	0.7519	0.912
*E. coli*	CARS	58	PLSR	0.9516	0.6528	0.8868	0.9956	0.7239	1.4035
			PCR	0.8745	1.0306	0.7429	1.4423	0.7721	1.2927
			BP	0.8209	0.8091	-	-	0.5847	1.3475
	SPA	19	PLSR	0.8888	0.9735	0.7800	1.3484	0.7587	1.3251
			PCR	0.8969	0.9395	0.7887	1.3246	0.7744	1.2870
			BP	0.8191	0.8126	-	-	0.7415	1.1146
	GA	28	PLSR	0.8212	1.2125	0.6891	1.5615	0.8075	1.200
			PCR	0.8016	1.2703	0.6829	1.5741	0.7471	1.3522
			BP	0.8423	0.7635	-	-	0.7624	1.0749

**Table 7:** Comparison of prediction models for surface TVC and *E. coli* values of mandarin fish.

Microbial Indicator	Location	Model	Number of Wavelengths	$R_{c}$	RMSEC	$R_{p}$	RMSEP
TVC	Back	RAW-PCR	420	0.8642	0.8914	0.8402	1.0600
		RAW-CARS-PLSR	47	0.9226	0.7541	0.8437	0.9512
	Abdomen	RAW-PCR	420	0.8871	1.0297	0.8181	1.2500
		RAW-CARS-PLSR	38	0.9437	0.5836	0.8464	0.9474
*E. coli*	Back	RAW-PCR	420	0.9115	0.7259	0.7706	1.0797
		RAW-CARS-PLSR	39	0.9309	0.8147	0.7632	1.4046
	Abdomen	RAW-PCR	420	0.9486	0.6724	0.7802	1.2725
		RAW-CARS-PLSR	58	0.9516	0.6528	0.7239	1.4035

**Table 8:** Comparison of TVC and *E. coli* value prediction models for mandarin fish surfaces based on texture analysis of characteristic bands.

Microbial Indicator	Location	Model	$R_{c}$	RMSEC	$R_{cv}$	RMSECV	$R_{p}$	RMSEP	ΔE
TVC	Back	PLSR	0.7288	1.1428	0.6458	0.5047	0.7104	1.3763	0.2335
		PCR	0.7324	1.1411	0.6471	0.5138	0.7168	1.3571	0.2160
		BP	0.6884	1.2851	0.6224	0.4564	0.6756	1.3387	0.0536
	Abdomen	PLSR	0.7448	1.3964	0.6672	0.5294	0.7276	1.4212	0.0248
		PCR	0.7535	1.4471	0.6728	0.5047	0.7104	1.2748	0.1277
		BP	0.7224	1.161	0.6268	0.4648	0.6818	1.3145	0.1535
*E. coli*	Back	PLSR	0.7015	1.4764	0.6037	1.6684	0.6943	1.5941	0.1177
		PCR	0.7221	1.4241	0.6418	1.3987	0.7097	1.4635	0.0394
		BP	0.6639	1.3250	0.6113	1.6329	0.6168	1.6011	0.2761
	Abdomen	PLSR	0.7193	1.4360	0.6151	1.6475	0.7024	1.5782	0.1422
		PCR	0.7549	1.3341	0.6646	1.3352	0.7401	1.3789	0.0448
		BP	0.7011	1.2464	0.6288	1.4485	0.6877	1.2957	0.0493

**Table 9:** Comparison of TVC and *E. coli* value prediction models for mandarin fish surfaces based on principal component image texture analysis.

Microbial Indicator	Location	Model	$R_{c}$	RMSEC	$R_{cv}$	RMSECV	$R_{p}$	RMSEP	ΔE
TVC	Back	PLSR	0.7244	1.3122	0.6423	1.5941	0.7024	1.3946	0.0824
		PCR	0.7318	1.3998	0.6654	1.517	0.7074	1.4122	0.0124
		BP	0.7469	1.1126	0.7055	1.6047	0.7242	1.1491	0.0365
	Abdomen	PLSR	0.7620	1.3954	0.6972	1.4582	0.7347	1.4047	0.0093
		PCR	0.7633	1.3323	0.6943	1.4378	0.7394	1.3875	0.0552
		BP	0.7705	1.0273	0.7054	1.2243	0.6681	1.0615	0.0342
*E. coli*	Back	PLSR	0.6901	1.2216	0.6349	1.4244	0.6801	1.3345	0.1129
		PCR	0.7079	1.2064	0.6383	1.4211	0.6831	1.3273	0.1209
		BP	0.7047	1.2408	0.6026	1.6422	0.6163	1.6153	0.3745
	Abdomen	PLSR	0.7472	1.1578	0.6502	1.5714	0.7243	1.2199	0.0621
		PCR	0.7635	1.1138	0.6896	1.4699	0.7459	1.2511	0.1373
		BP	0.7312	1.1386	0.6765	1.3105	0.6966	1.2799	0.1413

**Table 10:** PCR model results of TVC and *E. coli* value prediction in mandarin fish based on feature layer fusion.

Microbial Indicator	Location	*R_c_*	RMSEC	*R_cv_*	RMSECV	*R_p_*	RMSEP
TVC	Back	0.6966	0.9836	0.6170	1.2958	0.6819	1.0277
	Abdomen	0.7057	0.9302	0.6299	1.2714	0.6886	0.9887
*E. coli*	Back	0.7033	0.9349	0.6287	1.2748	0.6823	1.0269
	Abdomen	0.7078	0.9265	0.6335	1.2622	0.6954	0.9495

**Table 11:** PCR model results of TVC and *E. coli* value prediction of mandarin fish based on decision level fusion.

Microbial Indicator	Method	Location	*R_c_*	RMSEC	$R_{cv}$	RMSECV	*R_p_*	RMSEP
TVC	Direct consolidation	Back	0.8915	0.9675	0.8864	0.9721	0.8291	1.1061
		Abdomen	0.9036	0.9634	0.8932	0.9664	0.8218	1.2084
	The D-S theory	Back	0.9315	0.7469	0.8822	0.9337	0.8389	1.1651
		Abdomen	0.9401	0.7387	0.8903	0.9026	0.8443	1.1464
*E. coli*	Direct consolidation	Back	0.8885	0.9746	0.8576	1.0926	0.8116	1.2882
		Abdomen	0.8897	0.9699	0.8786	1.0146	0.8180	1.2501
	The D-S theory	Back	0.9315	0.7559	0.8871	0.9024	0.8398	1.1638
		Abdomen	0.9469	0.7443	0.8969	0.8995	0.8512	1.1224

## Data Availability

The original contributions presented in this study are included in the article. Further inquiries can be directed to the corresponding author.

## Abbreviations

The following abbreviations are used in this manuscript:

1st De	1st Derivative
BP	Backpropagation
BPA	Basic Probability Assignment
BBA	Basic Belief Assignment
CARS	Competitive Adaptive Reweighted Sampling
E. coli	Escherichia coli
GA	Genetic Algorithm
GLCM	Grey level co-occurrence matrix
MSC	Multiplicative scattering correction
PCA	Principal Component Analysis
PCR	Principal Component Regression
PLSR	Partial Least Squares Regression
R2	Coefficients of determination
RMSE	Root Mean Square Error
ROI	Region of interest
SNV	Standard normal variate transformation
SPXY	Sample set partitioning based on joint x–y distance
SVC	Support Vector Classification
TVC	Total viable count
VN	Vector Normalization
